# Targeted Metabolomic Analysis of Serum Fatty Acids for the Prediction of Autoimmune Diseases

**DOI:** 10.3389/fmolb.2019.00120

**Published:** 2019-11-01

**Authors:** Dimitris Tsoukalas, Vassileios Fragoulakis, Evangelia Sarandi, Anca Oana Docea, Evangelos Papakonstaninou, Gerasimos Tsilimidos, Chrysanthi Anamaterou, Persefoni Fragkiadaki, Michael Aschner, Aristidis Tsatsakis, Nikolaos Drakoulis, Daniela Calina

**Affiliations:** ^1^Department of Clinical Pharmacy, Faculty of Pharmacy, University of Medicine and Pharmacy, Craiova, Romania; ^2^Metabolomic Medicine, Health Clinic for Autoimmune and Chronic Diseases, Athens, Greece; ^3^E.INu.M, European Institute of Nutritional Medicine, Rome, Italy; ^4^The Golden Helix Foundation, London, United Kingdom; ^5^Laboratory of Toxicology and Forensic Sciences, Medical School, University of Crete, Heraklion, Greece; ^6^Department of Toxicology, Faculty of Pharmacy, University of Medicine and Pharmacy, Craiova, Romania; ^7^Department of Molecular Pharmacology, Albert Einstein College of Medicine, The Bronx, NY, United States; ^8^Research Group of Clinical Pharmacology and Pharmacogenomics, Faculty of Pharmacy, School of Health Sciences, National and Kapodistrian University of Athens, Athens, Greece

**Keywords:** metabolomics, total fatty acids, biomarkers, inflammation, autoimmune diseases

## Abstract

Autoimmune diseases (ADs) are rapidly increasing worldwide and accumulating data support a key role of disrupted metabolism in ADs. This study aimed to identify an improved combination of Total Fatty Acids (TFAs) biomarkers as a predictive factor for the presence of autoimmune diseases. A retrospective nested case-control study was conducted in 403 individuals. In the case group, 240 patients diagnosed with rheumatoid arthritis, thyroid disease, multiple sclerosis, vitiligo, psoriasis, inflammatory bowel disease, and other AD were included and compared to 163 healthy individuals. Targeted metabolomic analysis of serum TFAs was performed using GC-MS, and 28 variables were used as input for the predictive models. The primary analysis identified 12 variables that were statistically significantly different between the two groups, and metabolite-metabolite correlation analysis revealed 653 significant correlation coefficients with 90% level of significance (*p* < 0.05). Three predictive models were developed, namely (a) a logistic regression based on Principal Component Analysis (PCA), (b) a straightforward logistic regression model and (c) an Artificial Neural Network (ANN) model. PCA and straightforward logistic regression analysis, indicated reasonably well adequacy (74.7 and 78.9%, respectively). For the ANN, a model using two hidden layers and 11 variables was developed, resulting in 76.2% total predictive accuracy. The models identified important biomarkers: lauric acid (C12:0), myristic acid (C14:0), stearic acid (C18:0), lignoceric acid (C24:0), palmitic acid (C16:0) and heptadecanoic acid (C17:0) among saturated fatty acids, Cis-10-pentadecanoic acid (C15:1), Cis-11-eicosenoic acid (C20:1n9), and erucic acid (C22:1n9) among monounsaturated fatty acids and the Gamma-linolenic acid (C18:3n6) polyunsaturated fatty acid. The metabolic pathways of the candidate biomarkers are discussed in relation to ADs. The findings indicate that the metabolic profile of serum TFAs is associated with the presence of ADs and can be an adjunct tool for the early diagnosis of ADs.

## Introduction

The distinction between self and foreign is a tightly regulated process of the immune system, and defects in any of the participating mechanisms may lead to autoimmune diseases (ADs) (Menni et al., 2017). ADs incidence has increased dramatically over the last decades, currently affecting 50 million people in the US alone, especially at younger age (American Autoimmune Related Diseases Association, 2018). This rapid rise is possibly related to urbanization and higher socio-economic status, which have shifted nutritional preferences toward industrialized and low quality food with additives (Lerner et al., 2016). Twin studies unraveled key genetic determinants to ADs, especially for Major Histocompatibility Complex (MHC) haplotypes based on the findings that ADs concordance is higher in monozygotic twins (Theofilopoulos et al., 2017). However, familial association to genetic predisposition is higher in early-onset diseases suggesting that factors other than gene have an impact on ADs as well (Cooper et al., 1999; Gangemi et al., 2016; Negrei et al., 2016; Petrakis et al., 2017; Buha et al., 2018). In a recent review, the authors discuss the role of metabolic workload in immunological tolerance. Their proposed model suggests that chronic malnutrition, including high calories and nutrients intake for long periods, leads to the loss of tolerance through the generation of high pro-inflammatory T cells vs. the regulatory T cells that control inflammation (De Rosa et al., 2017). They propose that metabolic disturbance should be added to the hygiene model that has been applied to explain the rapid rise of chronic conditions (Bach, 2002). In addition, the World Health Organization (WHO) has suggested that the modifiable risk factors are the cause of chronic diseases in more than 80% of the cases (WHO, 2019). Modifiable factors are not presently satisfactorily considered within the standard medical approach (Strong et al., 2006; Tinetti et al., 2012).

Metabolomics can provide data for nutritional deficiencies, metabolic imbalance, environmental burden, and the gut microbiome. These factors can be modified through diet, lifestyle, supplements, and medication (Dahan et al., 2017). Key metabolic pathways, including the metabolism of glucose, protein and carbohydrates, fatty acids oxidation, mitochondrial function, neurotransmitters metabolism, and markers of oxidative stress and microbiome, are critically assessed through metabolomics (Lee et al., 2015). Quantification and evaluation of metabolites is the most effective method to capture time-dependent fluctuations and cellular metabolic state even prior to disease onset. Measurement of metabolites in patients with ADs and experimental ADs models have shown that there are significant metabolism fluctuations during the development of the disease (Leslie and Beyan, 2011; Hao et al., 2017). Findings from a randomized clinical trial on asthmatic children showed that urinary organic acids could be potential biomarkers to track the progression of the disease (Papamichael et al., 2018). Total fatty acids (TFAs) are valuable markers of inflammation and gain increasing attention in cases of chronic inflammation as in ADs (Serhan et al., 2007). We have previously reported the reference values of TFAs in a healthy Greek population, discussing the role of age, gender, diet, and measurement method of the levels of TFAs (Tsoukalas et al., 2019). Given these observations, we measured serum TFAs in patients with ADs using targeted GC-MS, aiming to identify an improved combination of these biomarkers as a predictive factor for the presence of ADs (Tsoukalas et al., 2019).

## Materials and Methods

### Subjects

A retrospective nested case-control study (Ernster, 1994) was conducted based on 5.850 subjects who visited the “Health clinic for autoimmune and chronic diseases” in Athens, Greece during the period of 3/8/2012 till 29/12/2017. All personal data were collected via the electronic platform of the clinic by trained administrative staff. The retrospective cohort study consisted of 1.950 patients for whom there were detailed records. A total of 240 patients with confirmed AD diagnosis were included in the present study, and 163 healthy individuals were assigned to the control group. Personal data of participants included age, gender, AD type, BMI, medical and nutritional history, and metabolomic analysis was performed in peripheral blood samples.

Exclusion criteria for the control group were obese (18.5<BMI<29.9), athletes, pregnant or lactating women, individuals taking any supplements and/or medication, and individuals diagnosed with a chronic or acute disease.

Inclusion criteria for the control group were adults 18–60, not taking any medication or supplements, and not suffering by any chronic or acute disease. Inclusion criteria for AD patients were individuals diagnosed with thyroid AD (THY), and/or inflammatory bowel disease (IBD), and/or psoriasis (PSO), and/or rheumatoid arthritis (RA), and/or vitiligo (VIT), multiple sclerosis (MS) and/or other AD (full list of other AD and comorbidities is available in Table S1).

RA diagnosis was based on ACR/EULAR 2010 Rheumatoid Arthritis Classification Criteria (Kay and Upchurch, 2012).

IBD: diagnosed according to the Lennard-Jones diagnostic criteria for Ulcerative colitis and Crohn's disease (Sherlock and Benchimol, 2017). PSO: Eligible PSO patients had to have chronic plaque type of PSO, and PASI score was used to assess the severity of the disease.

THY: As there are no international criteria for autoimmune thyroid disease classification diagnosis was performed according to levels of TSH, T3, and T4 and thyroid gland ultrasound to do disease classification.

VIT: Diagnosis performed according to Vitiligo Global Issues Consensus Conference (Kong et al., 2011).

MS: Diagnosis performed according to the McDonald 2010 diagnostic criteria (Polman et al., 2011).

Because the correlation of FA profiles to the clinical parameters of each AD is out of the scope of this paper, the clinical characteristics of patients for each AD group are not presented here but will be examined in separate studies. Also, it should be noted that in analyses such as we discuss here, matching of controls with cases is a commonly used method to control for confounding. However, there are several considerations concerning its proper use and, frequently, matching produces almost the same results with unmatching analysis or the gain in efficiency is modest. Nonetheless, for statistically exploratory purposes, we attempted to match case and controls concerning age and gender using the Propensity Score Matching (PSM) which has become a popular approach to estimate causal treatment effects (Rose and Laan, 2009; Faresjö and Faresjö, 2010). The analysis showed that there was any gain in terms of efficiency and, thus, we decided to conduct an unmatched analysis and adjusted any potential confounder via the multivariate analysis (Figure S3).

### Ethics Approval

All procedures performed in studies involving human participants were under the ethical standards with the 1964 Helsinki declaration and its later amendments, or comparable ethical standards. Participants of the study were informed that their personal data would be processed according to the EU General Data Protection Regulation (GDPR), and fully anonymization would be used for this study. Informed consent was obtained from participants. The study has been approved by the scientific board of the “Health clinic for autoimmune and chronic diseases” and the Ethics Committee of the University of Crete (approval no. A.P. 39_22112018).

### Chemicals

Methyl non-adecanoate (74208, Honeywell Fluka™; Honeywell, Seelze, Germany) was used as an internal standard. The calibration of the standard mixture was performed with a mixture of FA methyl esters (47885-U; Supelco-Sigma-Aldrich, St. Louis, MO, USA). All other solvents used were of the highest purity available [methanol, n-hexane (both from Merck KGaA, Darmstadt, Germany), HCl (301721] and 2,6-i-tert-butyl-4-methylphenol (BHT, B1378l) (both from Sigma-Aldrich).

### Sample Preparation

The participants fasted for 12 h before their visit to the clinic. The metabolomic analysis was performed in the patients' blood samples using standard methodology (Tsoukalas et al., 2017). Briefly, peripheral blood was collected, and samples were centrifuged at 1,500 × g at 4°C to isolate the plasma. The plasma specimens were stored at −20°C prior analysis for up to 24 h to ensure that the samples would not degrade. In the case of hemolysis of the blood samples, the blood collection was repeated.

The standard internal mixture (200 μl methyl non-adecanoate in hexane containing BHT) was added to the 100 μl plasma. The FAs were hydrolyzed and derivatized into methyl esters by the addition of 5% v/v methanolic HCl. Transmethylation was performed at 90°C for 60 min, and then the samples were brought to room temperature. The extraction of FA methyl esters was performed using hexane, and they were transferred to GC injection vials with a crimp cap.

As previously described, during the preparation of the samples lipid extraction prior to methylation was not included since with MS, the FAs can be directly identified in plasma without affecting the quantity or quality (Stellaard et al., 1990).

### Gas Chromatography-Mass Spectrometry

The carrier gas used was helium, and the injection volume was 1 μl per sample.

The analysis was performed on an Agilent 7890A gas chromatograph (GC) coupled to a 5975C mass detector (MS quadrupole), equipped with an electron ionization (EI) source, operating in positive mode (Agilent Technologies, Santa Clara, CA, USA). The FA methyl esters were separated using an HP-5 ms capillary column (30 m × 250 μm × 0.25 μm). The conditions used were as follows: initial oven temperature was 70°C, the ramp rate was 4°C/min, the final temperature was 290°C, held for 4 min and the acquisition was in the scan mode.

### Statistical Analysis

All analyses were undertaken using IBM SPSS 22 (IBM Corp., Armonk, N.Y., USA) software^1^ and the free R-project software (https://www.r-project.org). A chi-squared test with continuity correction was used to determine whether there is a significant association between gender and the presence of AD. In order to assess the normality of distributions for biomarkers, QQ-plots were applied for each one of them, while univariate analyses comparing differences between the means were conducted with a Mann–Whitney *U*-test (*P* < 0.05). We further conducted a multivariate analysis of variance (MANOVA) which affords in a richer use of the information contained in the dataset and explore the effect of factors on several response variables via simultaneous hypotheses tests. In particular, the method runs the analysis on a new variable which is a linear combination of dependent variables and, thus, taking into account the potential correlation between exploratory biomarkers in our case. As an additional step, a Bonferroni correction was conducted to limit the type I error which is the probability to wrongly reject the null hypothesis at expenses of Type II errors (false negative) (Vinaixa et al., 2012). Principal Component Analysis (PCA) was applied to decompose the data into a few new variables which correspond to a linear combination of the originals. PCA is a multivariate data analysis aiming to reduce the dimension of expression data with minimum information loss, to visualize the similarities between the biological samples and to capture the most of the variation in the data set (Jolliffe et al., 2016). Outliers, the points that are distant from their own neighbors in the data set, were analyzed using a straightforward approach aiming to create a frequency of the continuous variables in a graphic form. After the outliers had been identified, the data were screened for outliers due to administrative (typo errors), and none was found. At the end, the deletion or retention of each outlier was clinically assessed. There was not any deletion since the sample was considered representative without any irregular pattern and, thus, we did not run any analysis to reduce the influence of the outliers. Next, with a binary response variable (“with” and “without” the AD) as an outcome, we built a logistic regression model including as independent variables the set of the principal components. The estimation of the model parameters is expressed via odds ratios. Since, frequently, PCA represents only a preliminary analysis of the available data, we also estimated the straightforward binary logistic regression with all the biomarkers as independent variables based on the backward selection method. Backward selection is a step-wise regression method which starts with a full model consisting of all candidate predictor variables (biomarkers). Based on the probability of the likelihood-ratio statistic, a removal testing was conducted to identify these variables that will remain in the model as statistically significant, via an iteration process (Heinze et al., 2018).

As a supplementary analysis, we employed an artificial neural network (ANN) framework to identify these biomarkers which predict better the presence of an AD. A mathematical presentation of this technique is out of the scope of the article and can be found by the interested reader elsewhere (Margarita, 2002). In short, ANN's are a family of a flexible form of equations which are often used for statistical analysis and data modeling, in which their role is perceived as an alternative to standard non-linear regression techniques. A neural network consists of a series of the so-called neurons that are interlinked to form a network, while each one of the links has a weight associated with it. ANN has an input layer, one or more hidden layers, and the output layer. An activation function is employed in the input layer, but also to the output layer to determine the outcome of the model. Differences amongst observed and predicted outcomes reinforce the model to readjust their weights of independent variables until a predetermined convergence is attained (the so-called “training” of the model). It must be mentioned that the optimal combination between the neurons and the number of layers which must be employed, remains a scientifically open question and in the most of cases, a trial-and-error approach is conducted by the researchers. In the present analysis, a Multilayer Perceptron (MLP) feed forward neural network was used and trained with the error back propagation algorithm (Saduf, 2013). The number of neurons at the input layer was determined by the number of biomarkers used. A non-strict pre-selection of included variables was conducted based on the p-value provided by the straightforward logistic regression. We assumed two hidden layers to capture the non-linear nature of the model. A sigmoid activation function was employed in the output layer to estimate the probability of the presence (or not) of an AD as a binary outcome. Before the training of the model, all data were transformed through standardized rescaling. We separate our model to training data set, test set and holdout. Holdout or random subsampling is a technique to evaluate predictive models by partitioning the original sample into a training set to train the model, and a test set to evaluate it. Finally, Receiver Operating Characteristic (ROC) curve analysis was used to assess the accuracy of predictions based on sensitivity and specificity for all the above mentioned models (Hajian-Tilaki, 2013).

## Results

### Characteristics of Patients With Autoimmune Diseases

**Figure 1:**
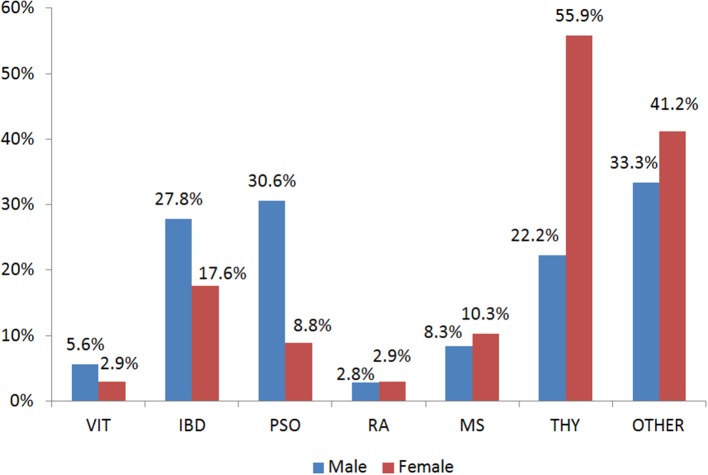
Gender Distribution of the Autoimmune Diseases in the case group. VIT, Vitiligo; IBD, Inflammatory Bowel Disease; PSO, Psoriasis; RA, Rheumatoid arthritis; MS, Multiple Sclerosis; THY, Thyroid autoimmune disease; OTHER, Other autoimmune disease.

In the present study, 403 participants were included; 240 patients with an AD (hereafter called case group) and 163 individuals in the control group. Among the patients, the majority had autoimmune thyroid disease (51.7%) while 29.7% of the total patients had more than one conditions (Table S1). Figure 1 depicts the percentages of ADs per gender for those belonging to the case arm. The baseline characteristics of the case and the control group are depicted in Table 1. Age was also not statistically significantly different between cases and controls (*p* > 0.05), while females represented the 70.2 and 63.6% for the case and control group, respectively. The Body-Mass Index (BMI), defined as weight in kilograms divided by the square of the height in meters, was estimated at 24.9 ± 4 for the control group, while was 25.4 ± 5 in the cases group, indicating that there was not a statistical difference in the 95% level of significance (*p* = 0.5). Concerning moderate physical exercise (3 times per week), 75.2% of total subjects answered positively in that question from the control group, while for the case group the corresponding percentage was limited to 56.7% (*p* = 0.011). Moderate alcohol consumption (3 glasses of wine per week) for those belonging to the case group and control group was 44.1 and 26.1%, respectively (*p* < 0.001).

**Table 1:** Baseline characteristics of the case and control group.

	**Case (*n* = 240)**	**Control (*n* = 163)**
^*^Age (Mean ± SD)	44.43 ± 11.4	43.3 ± 9.9
^*^Female (%)	70.2	63.6
^*^BMI (Mean ± SD)	25.4 ± 5	24.9 ±4
^a^^**^ Exercise n (%)	139 (56.7)	124 (75.2)
^b^^**^ Alcohol n (%)	108 (44.1)	43 (26.1)
THY n (%)	191(51.67)	0
RA n (%)	40 (10.81)	0
IBD n (%)	73 (19.7)	0
MS n (%)	32 (8.64)	0
PSO n (%)	62 (16.7)	0
VIT n (%)	15 (4)	0
OTHER n (%)	117 (31.67)	0

BMI, Body Mass Index; THY, Thyroid Autoimmune Disease; RA, Rheumatoid Arthritis; IBD, Inflammatory Bowel Disease; MS, Multiple Sclerosis; PSO, Psoriasis; VIT, Vitiligo;

a Exercise >3 times per week;

b alcohol consumption of 3 glasses of wine per week;

* p > 0.05;

** *p < 0.001*.

### Targeted Metabolomic Profiling of Patients With Autoimmune Diseases and Healthy Controls

**Figure 2:**
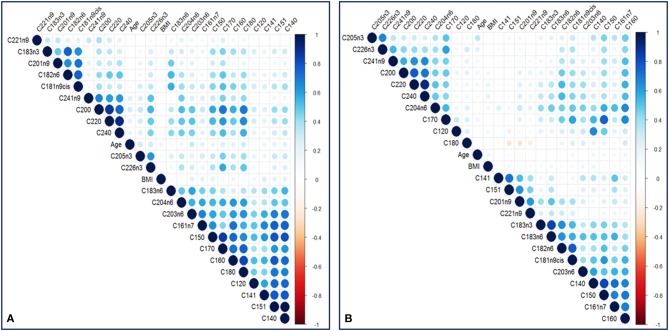
A scatter plot correlation matrix of the main variables used in the model. **(A)** Case group **(B)** Control group. Positive correlations are shown in blue and negative correlations are shown in red.

In total, 23 TFAs were tested on the available sample using GC-MS. Values of mean ± SD and median for each TFA, total omega-3, total omega-6, total Monounsaturated FA (MUFA), total Polyunsaturated FA (PUFA), total Saturated FA (SFA) for the two groups are listed in Table 2. The non-Parametric Mann-Whitney test was employed to detect differences among the variables in the two groups since Q-Q plots showed a deviation of normality. In total, 12 variables were statistically significant under the assumption of non-difference of distributions between the groups (Table 2). From the ratios included only total omega-6/total omega-3 was significantly different between the groups (*p* < 0.001). C22:6n3, total omega 3, C18:3n6, C15:1, C20:1n9, C12:0, C15:0, C17:0, C18:0 and total omega 6/ total omega 3 ratio were statistically significantly different between the two groups after Bonferroni correction. Correlation analysis was performed in the two groups to identify metabolite-metabolite correlations with age and BMI being included as variables. Specifically, in the case and control group, a total of 992 correlations were analyzed, among which 653 resulted in significant correlation coefficients in 90% level of significance (*p* < 0.05). Figure 2 depicts a scatter plot matrix showing the positive (blue) and negative (correlations) in the case group (left) and the control group (right). Overall, no statistically significant negative correlations were noted. Lauric acid (C12:0), pentadecanoic acid (C15:0), stearic acid (C18:0), myristoleic acid (C14:1), cis-10 pentadecanoic acid (C15:1) and arachidonic acid (C20:4n6) showed the strongest metabolite-metabolite correlations among the TFAs in the case group, while age was not correlated to any of these metabolites in any group.

**Table 2:** Concentrations of FA and FA ratios in case and control groups.

	**Case**		**Control**		
	**Mean ± SD**	**Median**	**Mean ± SD**	**Median**	***p*-value**
C183n3	14.6 + 6.4	13.8	13.3 + 10	12.2	0.006^*^
C205n3	50.6 + 35	40.3	52.4 + 74.2	35.7	0.297
**C226n3**	105.7 + 50.9	98.2	118.9 + 58.5	107.6	0.020
**Omega3**	178 + 82.2	163.6	218.5 + 129.6	194.2	<0.001^*^
C182n6	1597.2 + 667.9	1534.1	1540.7 + 573.3	1420.5	0.601
**C183n6**	24.7 + 21.1	18.6	18.6 + 15.7	14.8	0.001^*^
C203n6	107.7 + 44.5	102.1	103.8 + 40.9	100.1	0.558
C204n6	424.4 + 124.7	413.9	402.9 + 119.8	387.9	0.082
Omega6	2153.6 + 755.9	2052.5	2065.4 + 661.8	1937.9	0.313
C141	3.3 + 7.7	1.9	2.1 + 2.2	1.1	<0.001^*^
**C151**	28.4 + 19.9	23.3	14.6 + 18	6.6	<0.001^*^
C161n7	85.9 + 57.9	72.7	68.7 + 43.2	57.7	0.001^*^
C181n9cis	1001.6 + 437	936.6	984.3 + 425.1	886.2	0.516
**C201n9**	7.4 + 3.8	6.4	5.5 + 4.4	4.4	<0.001^*^
C221n9	1.5 + 0.8	1.4	1.5 + 2.1	1.0	<0.001^*^
C241n9	68.2 + 20	65.4	69.7 + 18.7	68.9	0.327
**C120**	8.1 + 10.9	5.2	11.9 + 11.7	7.2	<0.001^*^
C140	57.4 + 36.8	48.9	58.5 + 33.6	50.4	0.508
**C150**	12.5 + 4.8	11.6	13.3 + 5.2	12.8	0.119
C160	1740.6 + 551.3	1631.9	1617.8 + 363.8	1559.6	0.078
**C170**	16 + 4.8	15.2	17 + 4.8	16.8	0.026^*^
**C180**	516.2 + 137	502.7	560.5 + 127.5	558.9	<0.001^*^
C200	14.8 + 4.3	14.4	15.3 + 5.1	14.5	0.447
C220	37.9 + 11.4	37.6	38.4 + 10.8	36.5	0.700
C240	31.5 + 10	31.5	32.8 + 8.9	31.2	0.237
C204n6/ C205n3	11.5 + 6.3	10.3	11.2 + 5.7	10.1	0.901
C203n6/ C204n6	0.3 + 0.2	0.2	0.3 + 0.1	0.3	0.390
C18:2n6/ C20:3n6	17 + 9.6	14.6	16.6 + 7.9	15.7	0.677
**Omega6/ Omega3**	13.8 + 6.4	12.7	11.1 + 5	10.2	<0.001^*^
MUFA	1190.5 + 478.9	1155.8	1140.3 + 462	1029.8	0.188
PUFA	2330.7 + 784.6	2274.6	2282.9 + 702	2200.2	0.604
SFA	2433.5 + 719	2325.6	2313.1 + 522.3	2252.3	0.250
Total FA	5954.7 + 1713.6	5743.7	5735.8 + 1484.2	5538.0	0.197
BMI	25.4 + 5	25.2	24.9 + 4	24.4	0.502

*Non-Parametric Mann-Whitney test. Ho, The distribution of characteristics is the same between the groups. Concentrations of fatty acids are expressed as μmol/l*.

* *p < 0.05. Omega6, Total omega6 fatty acids; Omega3, Total omega3 fatty acids; PUFA, Polyunsaturated fatty acids; MUFA, Monounsaturated fatty acids; SFA, Saturated fatty acids. Bold indicates that the variables are considered statistically significant (p < 0.05) based on Bonferroni correction*.

**Figure 3:**
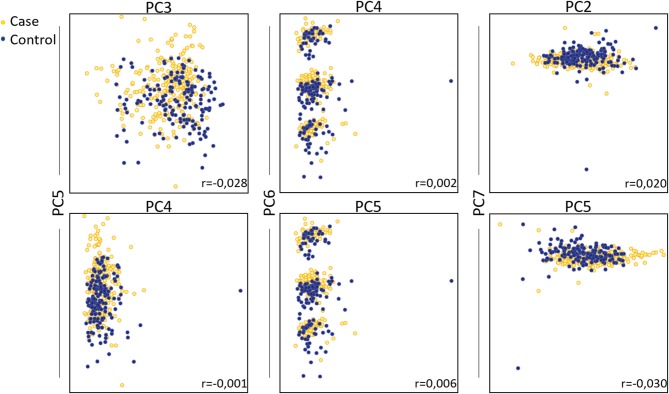
Principal component analysis on total fatty acids of patients with autoimmune diseases compared to control group. Pairwise score plots that the r coefficient was <0.030 are shown. Absolute r coefficient values are depicted in each plot.

Principal Component Analysis (PCA) was performed to visualize clusters within the samples. The data were screened for outliers due to administrative (typo errors), but none was identified. Due to the absence of missing data, all the available observations were included in the analysis. The Kaiser-Meyer-Olkin Measure of sampling adequacy for component analysis was estimated at 0.798, indicating reasonably well adequacy, while Bartlett's test of sphericity was statistically significant [*X*^2^ (253) = 5,102, *p* < 0.001]. Analysis indicated that the first seven components, which were based on the variables shown in table 1 with correlation coefficient <75%, explained in total 70.3% of the variance, while the rest of the components explained <4.5% of the total variance each. Hence, the seven-component solution, with eigenvalues >1, was preferred as a solution for the model (Table S2 and Figure S1). The component score coefficient matrix is depicted in Table 3. After the oblimin rotation, there was only a small correlation between each of the composite scores lower than 0.3 for all the components. Figure 3 depicts the combination of factors, which show lower correlations, and have *r* coefficient <0.030 in absolute values (pairwise score plots for components 1–7 in Figure S2).

**Table 3:** Component score coefficient matrix.

**Component**	**1**	**2**	**3**	**4**	**5**	**6**	**7**
C183n3	0.011	−0.005	−0.261	−0.028	−0.159	−0.052	0.189
C205n3	−0.003	−0.123	−0.011	0.500	0.041	−0.014	0.007
C226n3	−0.027	0.058	0.017	0.388	−0.015	0.008	0.048
C182n6	−0.050	0.045	−0.330	−0.036	−0.109	0.025	0.095
C183n6	0.007	−0.047	−0.303	−0.058	0.139	−0.088	0.008
C203n6	0.121	0.116	−0.017	−0.152	0.127	−0.064	−0.037
C204n6	0.076	0.132	−0.035	−0.015	−0.002	0.018	−0.092
C151	0.209	−0.128	−0.042	0.037	0.106	0.087	−0.081
C161n7	0.223	−0.064	0.023	−0.010	0.109	0.032	−0.167
C201n9	−0.049	−0.096	−0.319	0.132	0.069	0.110	−0.260
C221n9	0.062	0.023	0.034	−0.036	−0.163	−0.070	−0.682
C241n9	−0.065	0.261	0.068	0.109	0.047	0.018	−0.147
C120	0.137	−0.013	0.012	0.005	−0.284	−0.155	0.347
C140	0.227	−0.055	−0.009	0.010	−0.048	−0.035	0.095
C160	0.195	0.069	0.049	0.005	−0.063	0.045	−0.032
C170	0.069	0.085	−0.025	0.180	−0.089	−0.062	0.001
C180	0.112	0.178	0.107	−0.002	−0.132	0.014	0.199
C200	−0.011	0.272	0.011	−0.044	−0.028	−0.018	0.021
C240	−0.082	0.288	−0.079	−0.091	0.115	0.036	0.024
BMI	0.037	−0.004	0.007	−0.084	0.482	−0.058	0.091
Exercise	0.024	0.053	0.061	0.022	−0.275	0.559	−0.057
Alcohol	0.011	−0.002	−0.050	−0.024	0.146	0.691	0.101
Age	−0.024	0.047	0.037	0.141	0.455	0.031	0.051
% Variance	30,3	10,8	8,2	6,1	5,5	4,9	4,6
% Cumulative Variance	30,3	41,1	49,3	55,4	60,9	65,8	70,4

*Rotation Method: Oblimin with Kaizer Normalization; in the model used only variables with Spearman correlation coefficient <75%*.

### Association of TFAs and Autoimmune Disease

Table 4 shows a binary logistic regression model which was used to test the research hypothesis regarding the relationship between the likelihood that a patient will have an AD and components 1–7 and gender. The log of the odds of a subject being affected by an AD was negative related to component one, five, six, and gender (*p* < 0.001) and positive related to components two, three and seven (*p* < 0.001), while the association with component four was not statistically significant (Table 4). The Hosmer & Lemeshow (H-L) goodness of fit test was estimated at *X*^2^ (8) = 29,450, *p* < 0.001, while Nagelkerke (pseudo) *R*^2^ was 0.268. The model predicts correctly 86.7 and 57.1% of those without and with an AD, respectively. The overall predictive score was also 74.7%.

**Table 4:** Association of the presence of autoimmune disease with the Principal Components Dependent.

	**B**	**St Error**	**exp(B)**	**95% LCI**	**95% UCI**	***p*-value**
Factor 1	−0.285	0.141	0.752	0.570	0.992	0.044
Factor 2	0.578	0.132	1.783	1.376	2.310	0.000
Factor 3	0.570	0.136	1.769	1.354	2.310	0.000
Factor 4	0.107	0.114	1.113	0.891	1.390	0.348
Factor 5	−0.673	0.128	0.510	0.397	0.656	0.000
Factor 6	−0.294	0.118	0.745	0.591	0.940	0.013
Factor 7	0.328	0.130	1.389	1.076	1.792	0.012
Female	−0.595	0.253	0.551	0.336	0.905	0.019
Constant	−0.082	0.202	0.921			0.685

*Variable: Absence of autoimmune disorder; Binary Logistic Regression LCI: Lower Confidence Interval; UCI: Upper Confidence Interval*.

### Selection of TFAs as Distinctive Markers for Autoimmune Disease

**Figure 4:**
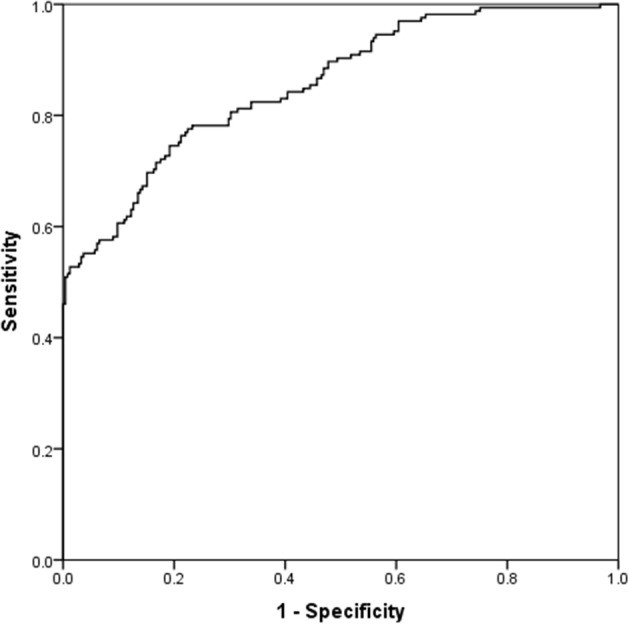
ROC curve for the straightforward binary logistic Model.

Table 5 presents the results for the straightforward binary logistic regression model. The model initially included all the variables with a correlation <0.75 based on backward selection, while only 6 of them were statistically significant in the new model. Alcohol abstinence, myristic acid (C14:0), and lignocericc acid (C:24:0) were positively correlated to the absence of an AD. Negative correlations with the absence of ADs were found in lack of exercise, cis-10 pentadecanoic acid (C15:1) and gamma-linolenic acid (C18:3n6). The (H-L) test was *X*^2^ (8) = 10,374, *p* = 0.240, while the Nagelkerke (pseudo) *R*^2^ was estimated at 0.556. Classification table indicates that the model predicts correctly 92.9 and 58.3.% of those with and without an AD, respectively. The overall predictive accuracy was 78.9%. ROC analysis indicates that the area under the curve was 0.856 (0.819–0.893), *p* < 0.001 (Figure 4).

**Table 5:** Association of the presence of autoimmune disease with patient's characteristics Dependent.

	**B**	**St Error**	**exp(B)**	**95% LCI**	**95% UCI**	***p*-value**
C183n6	−0.039	0.010	0.961	0.943	0.980	0.000
C151	−0.299	0.055	0.741	0.666	0.825	0.000
C140	0.154	0.027	1.166	1.106	1.230	0.000
C240	0.026	0.015	1.026	0.997	1.056	0.078
No exercise	−1.002	0.309	0.367	0.200	0.673	0.001
No alcohol	0.934	0.297	2.544	1.423	4.549	0.002
Constant	−1.847	0.528	0.158			0.000

*Variable: Absence of autoimmune disorder; Binary Logistic Regression Model; Stepwise Backward Method; Variable(s) entered on step 1: Exercise, Alcohol, Sex, C183n3, C205n3, C226n3, C182n6, C183n6, C203n6, C204n6, C151, C161n7, C201n9, C221n9, C241n9, C120, C140, C160, C170, C180, C200, C240, BMI*.

### Validation of the Distinctive Model for Prediction of Autoimmune Disease

**Figure 5:**
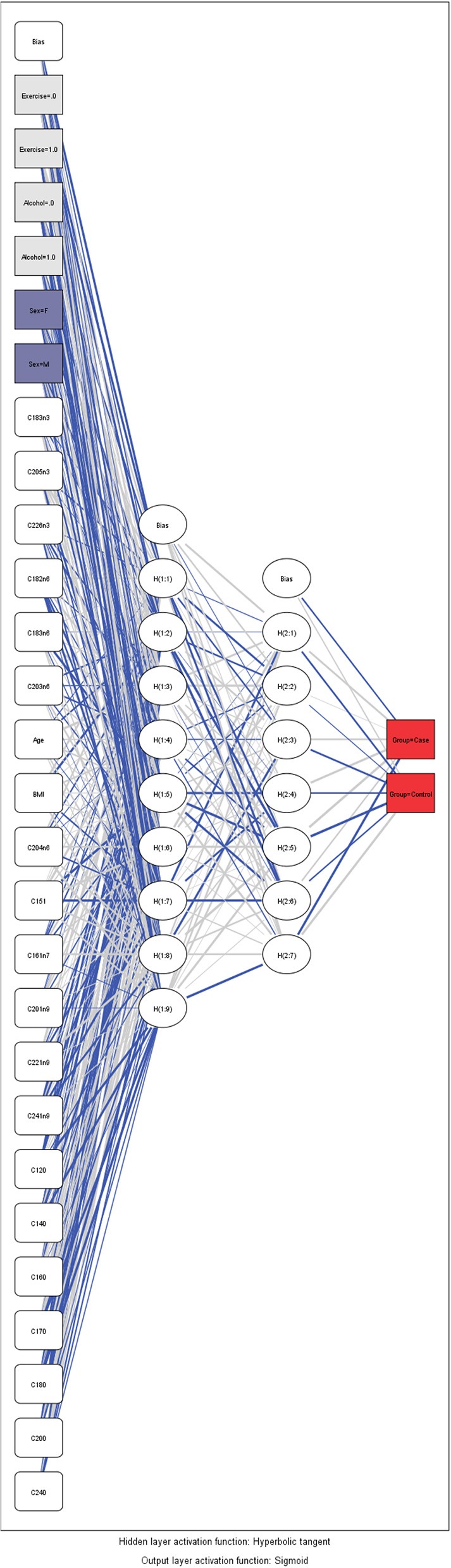
Architecture of the Artificial Neural Network.

**Figure 6:**
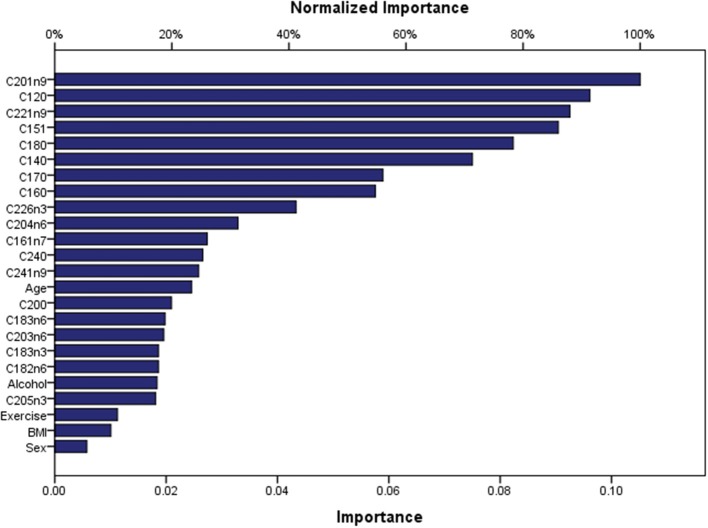
Contribution of biomarkers and factors to the predicted accuracy of the ANN.

Artificial Neuronal Networks (ANN) analysis was employed based on the architecture of the model presented in Figure 5. Initially, we adopted 2 layers of architecture, with all available variables in the input layer to describe the non-linear nature of our data set. Due to over-fitting and the relatively limited number of our observations, we reduced our model. In the end, a model with two hidden layers and 11 variables were employed in accordance with the previous logistic model. We used 273 (67.7%) observations as training data set, 88 (21.8%) observations as the test set, and 42 observations (10.4%) as a holdout. The parameters of the model are presented in Table S3. The overall predictive accuracy of the model was estimated at 76.2%. Total predictive value of the model is presented in Table 6. The area under the ROC curve was estimated at 0.792 for cases and controls. The most important biomarkers which contribute to the model were Cis-11-Eicosenoic (C20:1n9), Lauric acid (C12:0), Erucic acid (C22:1n9), Cis-10-pentadecanoic acid (C15:1), Stearic acid (C18:0), Myristic acid (C14:0), Heptadecanoic acid (C17:0), Palmitic acid (C16:0) (Figure 6).

**Table 6:** Classification table for artificial neural network.

	**Predicted**			
		**Case**	**Control**	**% Correct**
Training	Case	152	15	91.0%
	Control	41	65	61.3
	Overall percent	70.7%	29.3%	79.5
Testing	Case	50	4	92.6
	Control	16	18	52.9
	Overall percent	75.0%	25.0%	77.3
Holdout	Case	17	2	89.5
	Control	8	15	65.2
	Overall percent	59.5%	40.5%	76.2

## Discussion

In this study, we measured the levels of serum TFAs using targeted GC-MS metabolomics in patients with ADs and compared them to controls aiming to assess their potency as disease biomarkers. We hypothesized that metabolites are significantly altered in patients with ADs, including thyroid disease, rheumatoid arthritis, multiple sclerosis, vitiligo, psoriasis, and inflammatory bowel disease. In total, 28 biomarkers including 23 TFAs and demographic variables were measured in 403 individuals and data were analyzed using univariate analysis (chi-square, Man-Whitney test, and Wilkoxon Sign Rank test), as well as more advanced techniques, such as PCA analysis, logistic regression, and Artificial Neural Networks.

We found that AD patients had increased levels of C14:1, C15:1, C16:1n7, C20:1n9, C22:1n9, C18:3n3, C18:3n6, and total omega-6/ total omega-3 ratio while they had lower levels of total omega-3 fatty acids, C12:0, C17:0, C18:0 in a statistically significant manner (Table 2). However, Bonferroni correction indicated that only the levels of C22:6n3, total omega 3, C18:3n6, C15:1, C20:1n9, C12:0, C15:0, C17:0, C18:0 and total omega 6/ total omega 3 ratio were statistically significantly different. The high inter-correlations between metabolites may partially explain the different results obtained from Mann–Whitney test and Bonferroni correction. Indeed, the metabolite-metabolite correlation patterns were markedly different between the case and the control group indicating the metabolic re-programming of ADs in line with previous studies reviewed by Seeger et al. (Seeger, 2009; Amersfoort and Kuiper, 2017). Among the statistically significant correlations, lauric acid (C12:0), pentadecanoic acid (C15:0), stearic acid (C18:0), myristoleic acid (C14:1), cis-10 pentadecanoic acid (C15:1) and arachidonic acid (C20:4n6) were stronger correlated in the case than the control group (*p* < 0.001).

Three predictive models were built to estimate the probability of the absence of an AD as a function of gender, age, exercise, alcohol consumption, BMI, and TFAs as biomarkers. PCA analysis was used to reduce the representation of variables to only seven new artificial variables, and we created a new predictive model based on binary logistic regression. Furthermore, we estimated a straightforward logistic regression model with all 28 variables as potential independent biomarkers. In the end, only the statistically significant biomarkers were assessed by the model. As expected, the first model had slightly less accurate predictions (74.7 vs. 78.9%) compared to the second, since PCA reduces the portion of the information used from the initial data set. It needs to be mentioned that PCA analysis is mainly an exploratory technique aiming to investigate the data set at a first level. The main strength of this analysis –if any, depending on the data structure- is the reduction of dimension by creating artificial variables at expenses of accuracy (Jolliffe et al., 2016). However, the assumption that the principal components with highest variance will also contain the most information is a limitation of the analysis which is also observed in our study. Hence, the PCA plot does not show a considerable distinction between control and case groups and, thus, the factors of PCA do not seem sufficiently robust to be used for a satisfactory data interpretation. For the 78.9% predictive accuracy of the second model, myristic acid (C14:0), lignoceric acid (C24:0), Cis-10 pentadecanoic acid (C15:1), gamma-linolenic acid (C18:3n6), exercise and alcohol consumption were identified as the most sensitive markers. Exercise and alcohol are lifestyle variables that have a major impact on metabolism and the immune system directly. Several studies have discussed the beneficial role of physical activity not only in prevention but also for the improvement of disease progression and the quality of life of patients (Sharif et al., 2018). More importantly, it has been shown that regular moderate exercise can increase glucose uptake and reduce insulin resistance (DeFronzo et al., 1987). The role of alcohol consumption in health and its effects on the immune system have been extensively discussed, and although several studies show that moderate alcohol consumption may be beneficial to health (Carlé et al., 2012) others demonstrate that it has a detrimental effect on the gut microbiome and immunotolerance (Wang et al., 2010; Sarkar et al., 2015; National Institute on Alcohol Abuse and Alcoholism, 2000).

ANN analysis showed that the most important predictors for the ADs were the following: Cis-11-eicosenoic (C20:1n9), lauric acid (C12:0), Erucic (C22:1n9), Cis-10 pentadecanoic acid (C15:1), stearic acid (C18:0), myristic acid (C14:0), heptadecanoic acid (C17:0), palmitic acid (C16:0) in the order of importance. The predictive accuracy of the ANN model was comparable to the straightforward binary logistic regression (76.2%). These findings indicate that the metabolic pathways of SFAs and MUFAs are significantly affected in ADs. In the group of SFAs lauric acid (C12:0), myristic acid (C14:0), stearic acid (C18:0), lignoceric acid (C24:0), palmitic acid (C16:0) and heptadecanoic acid (C17:0) can be potent biomarkers. SFAs including stearic acid (C18:0), myristic acid (C14:0) and palmitic acid (C16:0) are endogenously converted to the MUFAs oleic acid (C18:1n9cis), myristoleic acid (C14:1) and palmitoleic acid (C16:1n7), respectively. This conversion is catalyzed by the Delta-9 desaturase, and the activity of the enzyme has been associated with insulin resistance (Kurotani et al., 2012), a key player in several Ads (Giles et al., 2015; Granata et al., 2017; Medina et al., 2018). Indeed insulin resistance has been linked to impaired desaturase activity and high levels of stearic and palmitic acid (Mayneris-Perxachs et al., 2014). Lignoceric (C24:0) is a very long chain fatty acid along with behenic acid (C22:0) and arachidic acid (C20:0). These are major components of ceramides that have been shown to have a protective role against insulin resistance and diabetes (Lemaitre et al., 2015). Heptadecanoic acid (C17:0) belongs to the odd-chain fatty acids, and although it has been widely used as a biomarker of dairy intake (Yakoob et al., 2014), there is recent evidence that it is related to metabolic diseases and gut microbiome imbalance (Jenkins et al., 2017). Insulin resistance is a common denominator in many chronic inflammatory diseases through complex molecular pathways (Engin et al., 2018). Because insulin inhibits lipolysis of stored fat, under insulin resistant conditions, free fatty acid levels increase in blood circulation and are taken up by organs that cannot store efficiently fat such as the liver and skeletal muscles. Excess fat in these tissues generates a cascade of mechanisms that lead to local insulin resistance and inflammation (Savage et al., 2005). From a different point of view, the Western diet has been implicated in the rapid rise of ADs as a result of multiple factors that break immunotolerance (De Rosa et al., 2017; Tsoukalas et al., 2019). Therefore, biomarkers that may predict and early diagnose insulin resistance would be very helpful in the prediction of chronic diseases.

In the case of MUFAs, Cis-10-pentadecanoic acid (C15:1), Cis-11-eicosenoic acid (C20:1n9) and erucic acid (C22:1n9) were demonstrated as potent biomarkers by our predictive model. Cis-11-eicosenoic acid (C20:1n9) originates from oleic acid (C18:1n9cis) and can be elongated to produce erucic acid (C22:1n9) (Bao et al., 1998). Erucic acid intake (through canola, Wallflower, or Lorenzo's oil) has been suggested to be beneficial for peroxisomal disorders like X-linked adrenoleukodystrophy by reducing the saturated VLCFA by negative feedback (Risé et al., 2014).

Gamma-linolenic acid (C18:3n6), the intermediate metabolite of linoleic acid (C18:2n6) conversion to dihomo-gama-linoleic acid (C20:3n6) and Arachidonic acid (C20:4n6) was also a sensitive marker for the predictive model. Dihomo-gama-linoleic acid (C20:3n6) and Arachidonic acid (C20:4n6) are the main precursors of the pro-inflammatory mediators. There have been several studies showing that arachidonic acid, along with other omega-6 and omega-3 fatty acids can be valuable markers in chronic inflammatory diseases because they reflect the inflammation status and the dietary preferences of the individual (Patterson et al., 2012; Tsoukalas et al., 2019). In a previous study, the authors demonstrated a strong relationship between serum fatty acid composition with the risk of type 1 diabetes-associated autoimmunity (Niinistö et al., 2017). A nested case-control analysis was performed within the Finnish Type 1 Diabetes Prediction and Prevention Study birth cohort, with 7,782 individuals. Fatty acids were associated with islet autoimmunity and primary insulin autoimmunity (higher palmitoleic acid, cis-vaccenic, arachidonic, docosapentaenoic, and docosahexaenoic acids decreased risk; higher α-linoleic acid and arachidonic: docosahexaenoic and omega-6/omega-3 acid ratios increased risk). The authors concluded that the fatty acid status might play a role in the development of type 1 diabetes-associated autoimmunity, but further studies are warranted to clarify the independent role of fatty acids in the development of type 1 diabetes.

A strength of this study is the application of ANN analysis to the targeted metabolomics data. ANN or logistic regression have been employed in many areas of health care research having advantages and disadvantages (Dreiseitl and Ohno-Machado, 2002). The most profound advantage of ANN is that it does not assume any pre-specified form of relationship between response and predictive variables but, on the contrary, the model itself investigates the relationship, which is not necessarily linear. Of course, due to this feature, ANNs have some disadvantages such as heavy mathematical computation and -more importantly- proneness to overfitting. Moreover, The ANN calculations represent a “data hungry” procedure and require an abundance of data to maximize its performance. In this light, it could be argued that the accuracy of our ANN model would have been even better in comparison with the binary logistic model if we had more data. However, this condition was not feasible for our study, but it is also not frequent in medical research, where dataset size is constrained by the complexity and the cost of large-scale experiments. As a general rule, it has been recommended that there should be approximately 10 times more training cases for each node of the model (Stathakis, 2009), but several statistical attempts have been made to reduce this numbers to smaller sample sizes (Pasini, 2015). Since our model has 9 nodes (at hidden layer 1) and 403 observations in total, this requirement is partially being fulfilled, including a fair sample size for an ANN analysis. In fact, an advantage of the present analysis is that it includes a relatively large sample of patients within the metabolomic context. As a general comment, beyond the ANN's requirements, the determination of a sample size per group is important in order to meet the criteria for a robust metabolomics analysis. Due to several complexities, there is currently no standard statistical methodology for this sample estimation (Nyamundanda et al., 2013; Trivedi et al., 2017). On a theoretical level, patient heterogeneity and other factors may play a role in the final estimation, but in practice, researchers usually include only 30–50 patients per treatment group, well below the number of subjects used in the present work.

One potential limitation of our study is that the cases (patients with ADs) had different diseases. Hence, this might be considered as a confounder and should be adjusted in statistical analysis, although this type of analysis requires a larger dataset. Our very next study will include additional variables of the population and focus on specific types of AD in order to explore more complex relationships between TFAs and pathogenesis of autoimmunity. Metabolomics is an emerging tool used for biomarker discovery as it can provide systemic understanding of the disease. However, there is some variability and inconsistencies among metabolomic studies, due to the experimental design (Kohler et al., 2017). This is a general characteristic of “omics” studies, since the small sample size results in limited statistical power especially when data require adjustment for multiple testing. In the present work, although the dataset outweighs the commonly used sample sizes, this limitation needs to be considered as the inclusion of volunteers affected by different diseases could hamper the interpretability of the results. However, many autoimmune diseases share common molecular mechanisms although different organs and cell types are involved, and, thus, probably have some common biomarkers (Arnald et al., 2016). Similarly, common biomarkers related to different diseases is observed even in a more “mature” field such as this of genomic medicine, a case that hampers the results interpretation as well (Fragoulakis et al., 2019).

The findings of the present study can be used as a baseline for studies on the metabolic fingerprint of ADs and highlight the potency of metabolomics and advanced statistical tools in prevention, prediction, treatment response, and drug side effects monitoring.

From this study, it can be concluded that the TFAs are associated with ADs presence, which is in line with the previous studies (Simopoulos, 2002). Overall, there is growing evidence that ADs have a distinct metabolic fingerprint which can be assessed through metabolomics, permitting a personalized approach and therapy. The metabolomic profile of TFAs can provide information regarding dietary intake and endogenous synthesized fatty acids. Thus, it needs to be critically assessed by the physician considering, the medical and nutritional history and the disease background as well (Trivedi et al., 2017). To this end, tailor-made interventions in nutrition and lifestyle might be of high therapeutic value in ADs.

## Data Availability Statement

The dataset presented in this study are available from the corresponding author upon reasonable request.

## Ethics Statement

The studies involving human participants were reviewed and approved by Ethics Committee of the University of Crete (approval no. A.P. 39_22112018) and Health Clinic for Autoimmune and Chronic Diseases. The patients/participants provided their written informed consent to participate in this study.

## Author Contributions

DT, AD, and DC conceived and designed the study and wrote the manuscript as a special part of a Ph.D. thesis. VF conducted the analysis, prepared the figures and tables and wrote the manuscript. ES interpreted the results, wrote the manuscript, and prepared the figures. EP supervised GC-MS experiments. GT and CA involved in collecting and managing the personal data of the participants and interpreted the results. AT and PF, involved in taking ethical clearance, critically assessed the design of the study and the manuscript. MA and ND wrote the manuscript and provided valuable comments in the discussion section. All authors reviewed the manuscript.

### Conflict of Interest

The authors declare that the research was conducted in the absence of any commercial or financial relationships that could be construed as a potential conflict of interest.

## Footnotes

^1^Available online at: https://www.ibm.com/analytics/spss-statistics-software
