# Supplementary material for: Targeted Metabolomic Analysis of Serum Fatty Acids for the Prediction of Autoimmune Diseases
Source: Front Mol Biosci. 2019 Nov 1;6:120. doi: 10.3389/fmolb.2019.00120 (PMC6839420; doi:10.3389/fmolb.2019.00120)
Supplement: Supplementary file 1 [file Data_Sheet_1.docx]

| **List of diseases** |
| --- |
| Asthma |
| Raynaud syndrome |
| Ankylosing spondylitis |
| Polyarthritis |
| Psoriatic arthritis |
| Scleroderma |
| Spoldyloarthritis |
| Osteoarthritis |
| Osteoporosis |
| Dermatitis - Eczema |
| Lupus |
| Autoimmune Polymyositis |
| Primary biliary cholangitis |
| Sjogren's syndrome |
| Alopecia areata |

**Table S1.** List of diseases grouped as “other” in the group of patients with autoimmune diseases.

| Component | Initial Eigen values | |  |
| --- | --- | --- | --- |
|  | Total | % of Variance | Cumulative % |
| 1 | 7.0 | 30.3 | 30.3 |
| 2 | 2.5 | 10.8 | 41.1 |
| 3 | 1.9 | 8.2 | 49.3 |
| 4 | 1.4 | 6.1 | 55.3 |
| 5 | 1.3 | 5.5 | 60.8 |
| 6 | 1.1 | 4.9 | 65.7 |
| 7 | 1.1 | 4.6 | 70.3 |

**Table S2.** Dimension Reduction with Principal Component Analysis

|  | Hidden Layer 1 | | | | | | | | | Hidden Layer 2 | | | | | | | Output Layer | |
| --- | --- | --- | --- | --- | --- | --- | --- | --- | --- | --- | --- | --- | --- | --- | --- | --- | --- | --- |
|  | (1:1) | (1:2) | (1:3) | (1:4) | (1:5) | (1:6) | (1:7) | (1:8) | (1:9) | (2:1) | (2:2) | (2:3) | (2:4) | (2:5) | (2:6) | (2:7) | Case | Control |
| (Bias) | 0.3 | -0.3 | 0.1 | 0.3 | 0.0 | -0.1 | 0.1 | 0.1 | 0.3 |  |  |  |  |  |  |  |  |  |
| [Exercise=.0] | 0.1 | 0.1 | -0.7 | 0.5 | -0.3 | -0.2 | -0.2 | 0.1 | -0.1 |  |  |  |  |  |  |  |  |  |
| [Exercise=1.0] | 0.5 | 0.1 | 0.1 | -0.5 | -0.3 | -0.3 | -0.1 | 0.7 | 0.1 |  |  |  |  |  |  |  |  |  |
| [Alcohol=.0] | -0.3 | 0.5 | 0.1 | 0.6 | -0.3 | 0.6 | -0.1 | 0.3 | 0.7 |  |  |  |  |  |  |  |  |  |
| [Alcohol=1.0] | 0.0 | 0.3 | -0.5 | -0.3 | 0.6 | 0.4 | 0.4 | -0.1 | 0.3 |  |  |  |  |  |  |  |  |  |
| [Sex=F] | 0.2 | 0.3 | -0.3 | 0.4 | -0.4 | 0.0 | 0.6 | -0.3 | -0.3 |  |  |  |  |  |  |  |  |  |
| [Sex=M] | -0.6 | 0.5 | -0.3 | -0.3 | 0.4 | -0.5 | -0.2 | -0.8 | 0.5 |  |  |  |  |  |  |  |  |  |
| C183n3 | -0.2 | -0.4 | 0.2 | -0.4 | 0.5 | 0.7 | -0.4 | 0.4 | -0.1 |  |  |  |  |  |  |  |  |  |
| C205n3 | -0.3 | -0.5 | 0.2 | 0.4 | 0.2 | 0.6 | 0.4 | -0.6 | 0.4 |  |  |  |  |  |  |  |  |  |
| C226n3 | -0.3 | -0.1 | -0.4 | 0.1 | -0.5 | -0.6 | -0.7 | 0.0 | 0.1 |  |  |  |  |  |  |  |  |  |
| C182n6 | -0.2 | -0.3 | -0.2 | -0.1 | -0.5 | 0.3 | 0.2 | 0.0 | 0.5 |  |  |  |  |  |  |  |  |  |
| C183n6 | -0.7 | 0.0 | 0.0 | -0.1 | -0.4 | -0.1 | 0.6 | 0.0 | 0.2 |  |  |  |  |  |  |  |  |  |
| C203n6 | -0.2 | -0.5 | -0.1 | 0.2 | -0.2 | -0.4 | -0.3 | -0.1 | 0.5 |  |  |  |  |  |  |  |  |  |
| Age | -0.1 | -0.7 | -0.1 | -0.2 | 0.3 | -0.5 | 0.0 | 0.3 | 0.0 |  |  |  |  |  |  |  |  |  |
| BMI | -0.3 | 0.4 | -0.1 | 0.1 | 0.2 | 0.3 | -0.4 | -0.4 | -0.1 |  |  |  |  |  |  |  |  |  |
| C204n6 | -0.7 | -0.1 | -0.3 | -0.2 | 0.0 | 0.2 | 0.4 | -0.6 | 0.3 |  |  |  |  |  |  |  |  |  |
| C151 | 0.1 | -0.7 | -0.8 | 0.1 | 0.8 | -0.1 | 1.0 | 0.2 | -0.1 |  |  |  |  |  |  |  |  |  |
| C161n7 | -0.1 | 0.0 | 0.2 | 0.3 | 0.2 | -0.1 | 0.2 | 0.6 | -0.3 |  |  |  |  |  |  |  |  |  |
| C201n9 | -0.3 | -0.4 | -0.7 | 0.4 | 1.0 | 0.3 | 0.6 | 0.3 | -0.8 |  |  |  |  |  |  |  |  |  |
| C221n9 | 0.1 | 0.2 | 0.3 | 0.5 | -0.2 | -0.6 | 0.0 | -0.2 | 0.2 |  |  |  |  |  |  |  |  |  |
| C241n9 | -0.4 | -0.2 | 0.1 | -0.1 | 0.5 | 0.6 | -0.3 | -0.3 | -0.6 |  |  |  |  |  |  |  |  |  |
| C120 | 0.4 | 0.0 | 0.9 | 0.2 | -0.4 | 0.4 | -0.3 | 0.3 | 0.4 |  |  |  |  |  |  |  |  |  |
| C140 | 0.7 | 0.0 | 0.4 | 0.3 | -0.3 | 0.5 | 0.0 | -0.4 | 0.3 |  |  |  |  |  |  |  |  |  |
| C160 | 0.1 | -0.6 | 0.0 | 0.7 | 0.5 | 0.6 | 0.2 | 0.4 | -0.5 |  |  |  |  |  |  |  |  |  |
| C170 | -0.3 | 0.1 | 0.1 | -0.5 | -0.2 | 0.4 | -0.6 | -0.4 | -0.4 |  |  |  |  |  |  |  |  |  |
| C180 | -0.2 | 0.4 | 0.1 | -0.6 | -0.2 | 0.2 | -0.6 | -0.4 | 0.5 |  |  |  |  |  |  |  |  |  |
| C200 | 0.2 | 0.2 | 0.3 | 0.4 | 0.2 | -0.3 | 0.3 | -0.1 | 0.3 |  |  |  |  |  |  |  |  |  |
| C240 | 0.4 | 0.2 | -0.5 | 0.1 | -0.5 | 0.2 | -0.2 | 0.4 | 0.6 |  |  |  |  |  |  |  |  |  |
| (Bias) |  |  |  |  |  |  |  |  |  | -0.5 | 0.2 | 0.1 | -0.1 | -0.3 | 0.5 | -0.6 |  |  |
| H(1:1) |  |  |  |  |  |  |  |  |  | 0.3 | -0.2 | -0.2 | -0.2 | -0.5 | -0.3 | -0.1 |  |  |
| H(1:2) |  |  |  |  |  |  |  |  |  | 0.4 | -0.1 | -0.4 | -0.2 | 0.0 | -0.7 | 0.0 |  |  |
| H(1:3) |  |  |  |  |  |  |  |  |  | 1.0 | -0.1 | 0.6 | 0.7 | 0.5 | -0.3 | -0.3 |  |  |
| H(1:4) |  |  |  |  |  |  |  |  |  | -0.1 | -0.6 | -0.6 | 0.5 | 0.3 | 0.3 | -0.4 |  |  |
| H(1:5) |  |  |  |  |  |  |  |  |  | -0.8 | -0.2 | -1.1 | -0.4 | -0.4 | 0.3 | -0.3 |  |  |
| H(1:6) |  |  |  |  |  |  |  |  |  | -0.1 | 0.2 | 0.1 | -0.7 | -0.1 | -0.2 | -0.1 |  |  |
| H(1:7) |  |  |  |  |  |  |  |  |  | -0.8 | 0.1 | -0.4 | 0.1 | -0.3 | 0.2 | -0.3 |  |  |
| H(1:8) |  |  |  |  |  |  |  |  |  | -0.1 | -0.1 | -0.4 | -0.2 | 0.5 | 0.3 | -0.1 |  |  |
| H(1:9) |  |  |  |  |  |  |  |  |  | 0.6 | -0.5 | -0.2 | 0.1 | 0.3 | -0.3 | 0.0 |  |  |
| (Bias) |  |  |  |  |  |  |  |  |  |  |  |  |  |  |  |  | 0.6 | 0.0 |
| H(2:1) |  |  |  |  |  |  |  |  |  |  |  |  |  |  |  |  | -1.4 | 1.3 |
| H(2:2) |  |  |  |  |  |  |  |  |  |  |  |  |  |  |  |  | -0.3 | 0.1 |
| H(2:3) |  |  |  |  |  |  |  |  |  |  |  |  |  |  |  |  | -0.3 | 0.2 |
| H(2:4) |  |  |  |  |  |  |  |  |  |  |  |  |  |  |  |  | -0.1 | 0.2 |
| H(2:5) |  |  |  |  |  |  |  |  |  |  |  |  |  |  |  |  | -0.2 | 0.2 |
| H(2:6) |  |  |  |  |  |  |  |  |  |  |  |  |  |  |  |  | 0.8 | -0.2 |
| H(2:7) |  |  |  |  |  |  |  |  |  |  |  |  |  |  |  |  | 0.0 | 0.1 |

**Table S3.** Model Parameters for the Artificial Neural Network


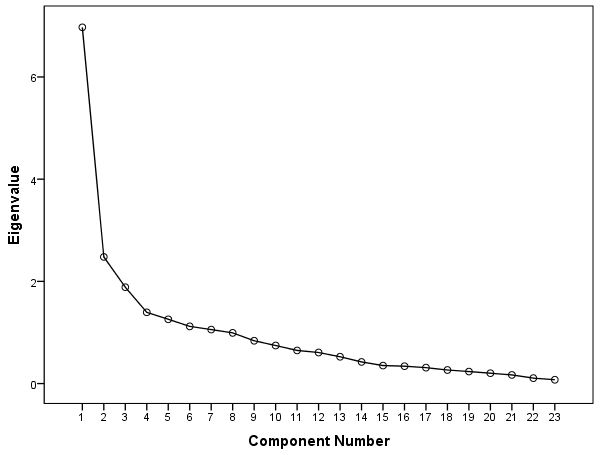


**Figure S1.** Graphical Representation of the Eigen-values greater than 1


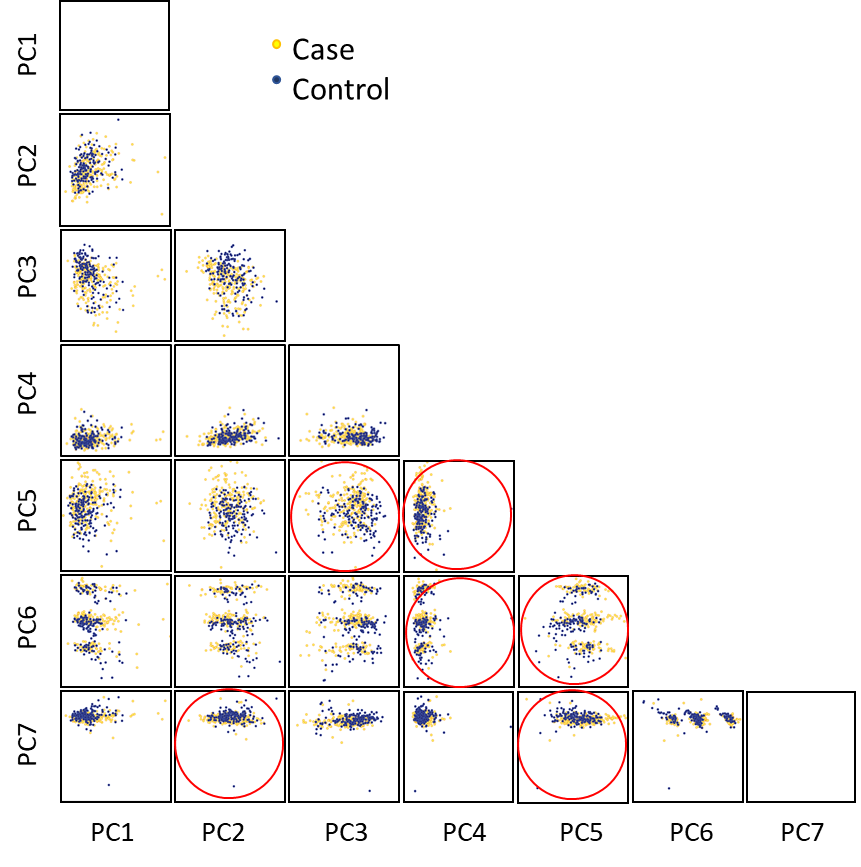


**Figure S2.** Graphical representation of principal component analysis factor.

Red circles indicate that r coefficient among factors is less than 0.0030 in absolute values.

**Figure S3.** Propensity Scores for matched and unmatched patients
